# Interindividual Variability in Functional Food Clinical Trials: Statistical Challenges, Responder Phenotypes, and Implications for Precision Nutrition

**DOI:** 10.3390/nu18213530

**Published:** 2026-10-27

**Authors:** Ryuji Takeda, Akiyoshi Sawabe

**Affiliations:** 1 Department of Nutritional Sciences for Well-Being, Faculty of Health Sciences for Welfare, Kansai University of Welfare Sciences, Osaka 582-0026, Japan; 2 Department of Applied Biological Chemistry, Faculty of Agriculture, Kindai University, Nara 631-8505, Japan; sawabe@nara.kindai.ac.jp

**Keywords:** precision nutrition, functional foods, interindividual variability, responders, non-responders, treatment-response heterogeneity, metabolomics, metabotyping, machine learning

## Abstract

Interindividual variability in response to dietary and functional food interventions is increasingly being recognized as a central issue in precision nutrition. However, individuals showing large pre–post changes are frequently classified as responders without adequately considering measurement error, within-person biological variability, regression to the mean, or distinction between the observed outcome variability and true heterogeneity in treatment effect. This review critically examines responder and non-responder concepts in functional food and dietary intervention trials, focusing on statistical interpretation, clinical trial design, biological determinants, metabolomics, metabotyping, multi-omics integration, and machine-learning approaches. We highlight the limitations of threshold-based responder classification, separate within-group significance testing, post hoc subgroup analyses, and high-dimensional predictive models without appropriate validation. Evidence from representative intervention studies is organized along a progression from retrospective responder classification and biological characterization to baseline predictor identification, predictive modeling, external validation, and prospective response-guided intervention. Particular attention is given to the distinction between explaining why individuals appeared to respond and predicting response before intervention. Based on the methodological and empirical evidence reviewed, we propose a structured hierarchy for responder research, ranging from descriptive heterogeneity to prospective response-guided intervention. The key challenge for precision nutrition is therefore not simply to identify who appeared to respond, but to determine whether heterogeneous responses are reproducible, treatment-specific, prospectively predictable, and useful for improving intervention selection. Moving from retrospective classification toward validated prospective prediction will be essential for translating responder research in functional foods into rigorous and clinically meaningful precision nutrition.

## 1. Introduction

Dietary and functional food interventions are generally evaluated according to their average effects at the population level. Randomized controlled trials are primarily designed to determine whether an intervention produces a statistically and clinically meaningful benefit when compared with an appropriate control condition. However, individuals exposed to the same dietary intervention frequently exhibit substantial differences in the magnitude and direction of observed responses [1,2,3,4].

Such interindividual variability has become increasingly important with the emergence of precision nutrition, which aims to move beyond population-average recommendations toward dietary strategies that account for individual biological, behavioral, and environmental characteristics. Large-scale studies on postprandial metabolism have demonstrated that responses to identical foods can vary markedly among individuals, and this variability can partly be predicted using combinations of clinical characteristics, habitual diet, lifestyle, gut microbiome composition, and other person-specific information [5,6,7].

Within this context, the terms “responder” and “non-responder” have become increasingly common in nutrition and functional food research. Participants may be classified according to changes in body weight, circulating lipids, glucose regulation, blood pressure, inflammatory biomarkers, gastrointestinal symptoms, cognitive outcomes, or other physiological and subjective endpoints [3,8,9]. Such classifications are intuitively attractive because they provide a direct route from heterogeneous trial outcomes to personalized dietary recommendations. However, responder-related analyses in functional food research are vulnerable to several common methodological problems. These include arbitrary or data-driven response thresholds, failure to account for measurement error and within-person biological variability, regression to the mean, inappropriate reliance on separate within-group significance tests, post hoc subgrouping, multiplicity, and lack of independent validation. Such issues can create or exaggerate apparent responder phenotypes and may lead to overinterpretation of observed heterogeneity as evidence of true treatment-response differences. A rigorous responder framework must therefore distinguish descriptive variability from reproducible treatment-response heterogeneity and from prospectively validated prediction. However, interpretations of responder and non-responder phenotypes are considerably more complex than they initially appear.

An observed change in an individual reflects not only the causal effect of the intervention but also measurement error, spontaneous biological variation, regression to the mean, adherence, temporal changes in lifestyle, and other uncontrolled influences [1,2,3,4]. Consequently, a participant showing a notable improvement during one intervention period is not necessarily a reproducible responder, whereas an individual showing little observed change cannot automatically be considered biologically nonresponsive.

Therefore, the statistical distinction between variability in observed outcomes and true heterogeneity in treatment effects is fundamental. A wide variation in change scores within an intervention group does not demonstrate that individuals differ in their causal responses to treatment [2,8]. Similarly, dividing continuous responses into responder and non-responder categories may introduce information loss, arbitrary threshold effects, and potentially misleading estimates of responder prevalence [3,10].

Existing reviews have addressed the individual components of this problem, including treatment-response heterogeneity, metabotyping, interindividual responses to plant bioactives, and precision nutrition [8,9,11,12,13,14,15]. However, an integrated framework linking the statistical validity of responder classification with biological characterization, prospective prediction, independent validation, and response-guided implementation in functional food trials remains limited.

These issues are particularly relevant in functional food research. Expected effects may be modest relative to biological variability, and trials frequently include multiple physiological and molecular endpoints in comparatively small study populations [4,8,9]. Therefore, exploratory responder analyses may generate biologically informative hypotheses while remaining vulnerable to statistical instability and limited reproducibility.

At the same time, advances in metabolomics, gut microbiome profiling, genomics, transcriptomics, digital phenotyping, and machine learning provide new opportunities to investigate the basis of heterogeneous responses [11,12,16,17]. Metabolomics and metabotyping are particularly relevant because they provide dynamic information reflecting the combined influence of dietary exposure, endogenous metabolism, microbial activity, and physiological state [11,12].

Nevertheless, the molecular characterization of individuals who responded in a completed trial is fundamentally different from demonstrating that the response can be predicted before intervention. Similarly, strong predictive performance within a development dataset does not establish whether a model generalizes to independent populations or improves intervention selection in practice [18,19,20].

Representative dietary and functional food intervention studies illustrating different stages of responder research are summarized in Table 1, and the inferential scope and limitations of commonly used responder-analysis approaches are outlined in Table 2.

This review critically examines responder and non-responder concepts in functional food and dietary intervention trials, with an emphasis on statistical validity, clinical trial design, biological determinants, responder phenotyping, and prospective response prediction. Attention is given to metabolomics, metabotyping, multi-omics integration, and machine-learning approaches. Finally, we propose a structured framework distinguishing descriptive heterogeneity, responder classification, biological characterization, prediction, independent validation, and prospective response-guided implementation.

Rather than asking simply who appeared to respond, the central question is whether an apparent response is reproducible, treatment-specific, prospectively predictable, and ultimately useful for precision nutrition.

## 2. Review Methodology

This article was designed as a structured narrative review with a critical methodological focus rather than as a systematic review or meta-analysis. The objective was to integrate evidence derived from statistical methodology, clinical trial design, functional food and dietary intervention studies, metabolomics and metabotyping, and emerging multi-omics and machine-learning approaches relevant to interindividual variability in nutritional responses.

The literature search was conducted in PubMed from database inception through 5 September 2026, using three complementary search strategies. The first strategy focused on responders, non-responders, interindividual variability, and treatment-response heterogeneity in nutritional and dietary interventions. The second focused on metabolomics, metabotyping, and gut microbiome approaches for responder identification and response prediction. The third focused on multi-omics, machine learning, artificial intelligence, and predictive modeling in precision and personalized nutrition. Search terms included combinations of “responder,” “non-responder,” “treatment response heterogeneity,” “interindividual variability,” “individual treatment response,” “functional food,” “dietary intervention,” “precision nutrition,” “personalized nutrition,” “metabolomics,” “metabotyping,” “microbiome,” “multi-omics,” “machine learning,” and “prediction.” Searches were restricted to English-language publications.

As several influential methodological studies relevant to individual treatment responses were not specific to nutrition and, therefore, could not be reliably identified using nutrition-focused search terms alone, the reference lists of key methodological reviews and eligible original studies were additionally screened. Selective forward citation searching of influential methodological and precision-nutrition studies was also performed to identify relevant publications that were not directly captured by the database searches.

Priority was given to (1) methodological studies addressing the statistical interpretation of individual treatment responses; (2) randomized or controlled dietary intervention studies explicitly evaluating responder/non-responder phenotypes or heterogeneous treatment responses; (3) studies investigating pre-intervention predictors of subsequent nutritional responses; (4) metabolomics, metabotyping, microbiome, or multi-omics studies relevant to responder identification or prediction; and (5) studies incorporating internal, external, or prospective validation of individualized response prediction.

Studies were considered eligible when they addressed at least one of the following: (1) statistical or methodological evaluation of interindividual treatment responses; (2) responder/non-responder classification or treatment-response heterogeneity in human dietary or functional food interventions; (3) pre-intervention predictors or modifiers of nutritional response; (4) metabolomics, metabotyping, microbiome, multi-omics, or machine-learning approaches relevant to response characterization or prediction; or (5) validation or prospective testing of individualized response prediction. Studies were excluded when they focused exclusively on population-average treatment effects without relevance to interindividual variability, when they were unrelated to nutritional or treatment-response methodology, or when they were not published in English. Animal and in vitro studies were generally excluded unless they were required to clarify a methodological or biological concept directly relevant to the interpretation of human responder research. As the aim of this review was conceptual and methodological with no intention to estimate a pooled intervention effect, study selection was based on relevance to the analytical framework rather than exhaustively including all nutritional intervention studies reporting variable responses. Representative studies were selected to illustrate the different stages of evidence development, including post hoc responder classification, biological characterization, baseline predictor identification, predictive modeling, external validation, and prospective response-guided intervention.

No formal meta-analysis or quantitative risk-of-bias assessment was performed because of the substantial heterogeneity in interventions, outcomes, responder definitions, analytical approaches, and study objectives.

**Figure 1:**
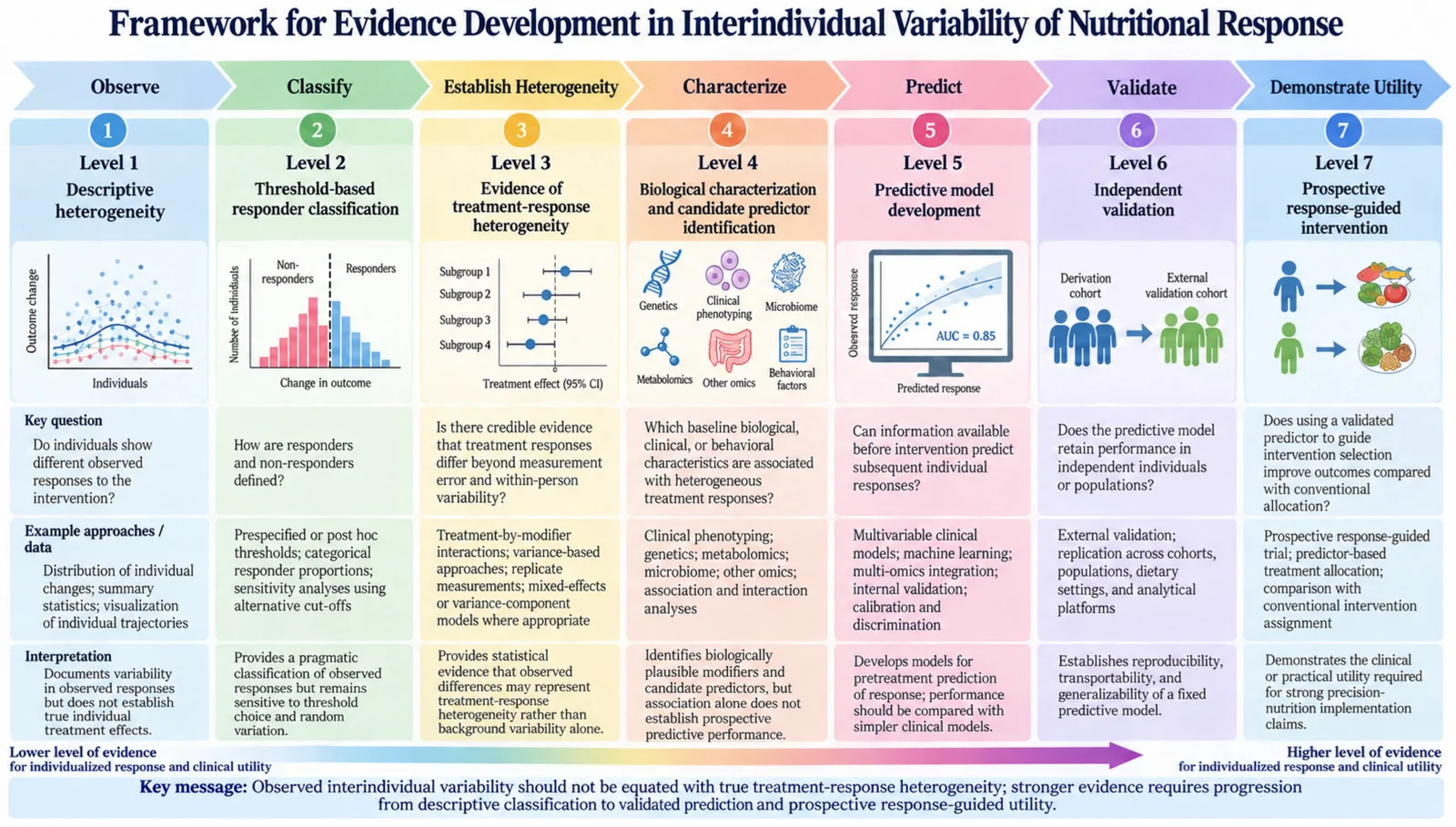
Framework for evidence development in interindividual variability of nutritional response. The proposed framework illustrates progression from descriptive heterogeneity and threshold-based responder classification to formal evaluation of treatment-response heterogeneity, biological characterization and candidate predictor identification, predictive model development, independent validation, and prospective response-guided intervention. Higher levels represent increasingly rigorous evidence that observed variability reflects reproducible and prospectively useful differences in treatment response and therefore provide stronger support for precision-nutrition implementation.

During manuscript preparation, ChatGPT (OpenAI, San Francisco, CA, USA) was used to assist with manuscript organization, language refinement, and development of the conceptual illustration presented in Figure 1. All scientific interpretation, literature evaluation, reference verification, and conclusions were independently determined and verified by the authors.

## 3. Concepts and Definitions of Responders and Non-Responders

### 3.1. What Constitutes a Response?

The terms “responder” and “non-responder” are widely used in clinical and nutritional research, yet their definitions vary considerably across studies [3,8]. Simply, a responder is an individual who demonstrates a predefined degree of improvement in a selected outcome following an intervention, whereas a non-responder does not meet this criterion. However, classification depends on several methodological choices, including the selected outcome, magnitude and direction of change, timing of assessment, and whether the threshold is statistically, biologically, or clinically defined [3,4].

A response may be expressed as an absolute change, percentage change, attainment of a target value, or movement beyond a predefined threshold. These approaches are not interchangeable and may yield substantially different estimates of responder prevalence. Accordingly, the response should generally be regarded as an operational definition rather than an intrinsic biological property unless reproducibility has been demonstrated [1,2].

### 3.2. Absolute and Relative Changes

Response is frequently defined according to the absolute or percentage change from baseline. Absolute changes remain directly interpretable in the original units, whereas relative changes may facilitate comparisons among individuals with different baseline levels. However, percentage changes may become unstable when baseline values are small and can strengthen the dependence on baseline measurements [21]. Neither approach establishes whether the observed change represents a reproducible treatment effect rather than a biological or analytical variation [2,4].

### 3.3. Threshold-Based Definitions

Responder classifications may use thresholds based on clinical guidelines, reference values, minimal important differences, analytical variations, or investigator-defined percentage changes. Threshold-based definitions are useful when an intervention aims to achieve a clearly interpretable physiological or clinical target. However, individuals immediately above and below a cutoff may be biologically similar despite being assigned to different categories [10]. Therefore, responder status should complement rather than replace the analysis of underlying continuous responses [10,22].

### 3.4. Clinically Meaningful Change

Minimal clinically important differences are valuable when validated thresholds exist. However, many outcomes used in functional food research, including inflammatory, oxidative stress, postprandial, and exploratory metabolic biomarkers, lack universally established clinically meaningful thresholds. Therefore, a statistically detectable change may not represent a meaningful health benefit. Statistical detectability, biological relevance, and clinical importance should be distinguished.

### 3.5. Statistical, Biological, and Clinical Response

For conceptual clarity, responder status may be considered at several levels. A statistical response may represent a change beyond expected measurement uncertainty or within-person variability [4]. A biological response may represent a reproducible physiological or molecular change consistent with the proposed mechanism. A clinical response may represent a meaningful improvement in terms of symptoms, function, or health risk. These concepts may overlap but are not equivalent.

### 3.6. Single-Outcome and Composite Definitions

Responder status is commonly based on a single primary outcome, although nutritional interventions can affect multiple physiological domains. Composite definitions may incorporate several variables; however, they introduce additional decisions regarding variable selection, weighting, correlation, and the required magnitude of improvement. Therefore, such definitions should be prespecified and justified [18,22].

### 3.7. Response as a Context-Dependent Phenotype

Responder status may depend on the dose, intervention duration, baseline metabolic state, habitual diet, medication use, adherence, and endpoints evaluated [1,8,9]. An individual classified as a non-responder under one condition may respond under another. Therefore, the responder and non-responder labels should be regarded as context-dependent rather than permanent personal attributes.

### 3.8. Transparent and Reproducible Definitions

Studies should report the exact response outcome, timing, threshold, rationale, prespecification status, and whether measurement error or within-person variability was considered [3,4,22]. Continuous response distributions should also be reported whenever possible [10]. Importantly, defining a responder does not itself demonstrate true interindividual differences in treatment effects [2,8].

## 4. Statistical Challenges in Responder Classification

### 4.1. Observed Variability Does Not Necessarily Represent True Treatment-Response Heterogeneity

Substantial variation is often observed among participants receiving the same intervention. However, variation in the observed change should not automatically be interpreted as true heterogeneity in treatment response. Observed changes reflect treatment effects together with measurement errors, spontaneous biological variations, and other within-person fluctuations [2,4].

Senn emphasized that treatment response cannot necessarily be assumed to represent a stable individual characteristic based on limited observations [1]. Atkinson and Batterham further demonstrated that considerable variation in observed change scores may occur even when all participants have the same true treatment effect [2]. Therefore, the distribution of individual changes within an intervention group alone provides insufficient evidence of true response heterogeneity.

### 4.2. Importance of an Appropriate Comparator

Potential treatment-response heterogeneity can be evaluated by comparing variability in change under active intervention with the corresponding variability under control conditions [2]. Greater variability under active intervention may be compatible with treatment-by-individual interaction, whereas similar variability under active and control conditions suggests that background within-person variability may account for much of the observed heterogeneity [2,8]. However, an excess of post-intervention or change-score variance in the active group should not, by itself, be interpreted as evidence of true treatment-response heterogeneity. Variance differences may also arise from baseline-dependent heteroscedasticity, differential or selective dropout, measurement properties, or other non-treatment-related sources of variability. Variance-based comparisons should therefore be regarded as screening evidence for possible heterogeneity rather than direct proof of individual treatment effects. Where the study design and repeated measurements permit, these comparisons should be complemented by appropriately specified mixed-effects or variance-component models that estimate between-individual variation in treatment effects while accounting for within-person variability.

### 4.3. Measurement Error and Within-Person Variability

Outcome reliability places a fundamental limit on responder classification. Repeated baseline and post-intervention measurements, standardized measurement procedures, and estimates of analytical and biological variation may improve individual-response assessments [4,23]. An observed change should not be interpreted as a meaningful individual response if its magnitude is indistinguishable from the expected measurement or biological variability.

### 4.4. Regression to the Mean and Baseline Dependency

Regression to the mean is particularly relevant when participants are selected based on elevated or reduced baseline values [2,21]. Extreme baseline measurements may become less extreme on repeat measurements even in the absence of treatment. Therefore, associations between baseline values and subsequent changes require cautious interpretation.

### 4.5. Problems Associated with Dichotomization

Continuous treatment responses are frequently converted into binary responder categories. However, dichotomization reduces information and statistical power while creating artificial distinctions between individuals immediately above and below a defined cutoff [10]. A continuous response should generally be the primary analytical scale, with categorical responder analyses reserved for situations in which thresholds are clinically, biologically, or analytically justified.

### 4.6. Comparing Responder Proportions

A difference in responder prevalence observed between intervention groups does not necessarily establish true interindividual heterogeneity in treatment effect. As mean treatment effects and random variability can influence the probability of crossing a defined threshold [3], responder proportions should be interpreted alongside continuous outcomes and comparator-group variability [2,3].

### 4.7. Subgroup Analyses and Interaction Tests

A statistically significant treatment effect observed in one subgroup and a nonsignificant effect observed in another does not demonstrate that the treatment effects differ between subgroups. Statistical significance depends not only on the magnitude of the estimated treatment effect but also on sample size, variability, and precision; therefore, two subgroup-specific estimates may differ in significance status even when their underlying treatment effects are similar. Conversely, both subgroup estimates may be statistically significant while still differing meaningfully in magnitude. To assess whether treatment effects genuinely differ across subgroups, the relevant inferential test is a formal treatment-by-subgroup or treatment-by-modifier interaction rather than comparison of separate subgroup-specific *p*-values. Such interaction analyses are often underpowered and vulnerable to multiplicity, particularly when conducted post hoc, and should therefore be interpreted cautiously.

### 4.8. Multiple Testing and Overfitting

Omics-based responder studies may evaluate hundreds or thousands of candidate variables using relatively small sample sizes. Post hoc response classification followed by high-dimensional feature selection creates substantial risks of false-positive results and overfitting [11,18,20]. Therefore, multiple-testing control, appropriate resampling, penalized modeling (where appropriate), and independent validation are essential.

### 4.9. From Outcome Variability to Treatment-Effect Heterogeneity

Heterogeneity in observed outcomes and in causal treatment effects are fundamentally different concepts [8,18]. Rigorous analysis should progress from measurement reliability and comparator-based assessment of variation toward the evaluation of treatment-response heterogeneity before attempting responder classification or prediction. The inferential implications of commonly used approaches are summarized in Table 2.

## 5. Clinical Trial Designs for Evaluating True Individual Responses

### 5.1. Conventional Parallel-Group Trials

Parallel randomized controlled trials are well suited for estimating average treatment effects but less suited for estimating reproducible individual treatment effects [8,24]. Each participant generally receives only one treatment condition, making the separation of true individual responses from within-person variability challenging.

### 5.2. Limitations of Single-Arm and Within-Group Pre–Post Comparisons

A statistically significant pre–post change within a single intervention group demonstrates only that an outcome changed over time; it does not establish whether the intervention caused the change [21]. Regression to the mean, temporal biological variations, lifestyle changes, expectancy effects, seasonal influences, and measurement errors may all contribute to the observed change [2,21].

Similarly, within a randomized trial, a significant pre–post change in the active group and nonsignificant change in the control group does not demonstrate a treatment effect. The relevant inferential question is whether the treatment groups differ directly [21,22]. Baseline-adjusted analysis of covariance, direct between-group contrasts, or appropriately specified treatment-by-time models are preferable.

### 5.3. Repeated Baseline and Post-Intervention Measurements

Repeated measurements can improve precision and provide estimates of within-person variability [4,23]. However, repeated measurements under a single intervention condition cannot reveal how the same participant would have responded under an alternative treatment.

### 5.4. Crossover Trials

Crossover designs allow each participant to receive both the active and control conditions, thereby improving within-person comparisons [8,24,25]. However, conventional single-cycle crossover still includes within-person random variability and generally provides limited evidence regarding reproducible person-by-treatment interactions. The period, sequence, carry-over, and washout effects must also be considered.

### 5.5. Repeated Crossover and Replicate Designs

Repeated exposure to active and control conditions provides stronger evidence for stable individual treatment effects because replicate crossover designs can separate person-by-treatment variability from within-person random variation [1,2,8,25]. Therefore, such designs are particularly informative when the objective is to establish reproducible treatment-response heterogeneity rather than merely an average within-person treatment contrast [25]. However, their principal limitations include participant burden, study duration, and the requirement for suitable washout periods. In nutritional interventions, additional challenges arise because whole foods and dietary patterns may be difficult to blind owing to differences in taste, texture, aroma, appearance, or meal structure. Sensory-matched control foods should therefore be used whenever feasible. Moreover, washout periods should be justified according to the expected persistence of the intervention effect, because some nutritional interventions may induce prolonged changes in the gut microbiota, tissue fatty acid composition, nutrient stores, or other physiological states that could carry over into subsequent treatment periods.

### 5.6. N-of-1 Trials

*N*-of-1 trials involve repeated randomized treatment and control periods within an individual, and they provide a rigorous approach to estimating individualized treatment effects when the intervention effects are sufficiently rapid and reversible [24]. Their validity similarly depends on adequate washout and, where blinding is intended, on the feasibility of providing a credible sensory-matched comparator. Digital monitoring and remote data collection may improve the feasibility of these approaches in nutritional research.

### 5.7. Run-In Periods

Run-in periods may standardize background conditions, assess baseline stability, and confirm adherence. However, excluding unstable or poorly adherent participants during the run-in may reduce external validity.

### 5.8. Enrichment Designs

Enrichment designs recruit individuals who are considered more likely to benefit according to prespecified or validated characteristics. These approaches may improve trial efficiency but depend on credible prior evidence that the enrichment variable modifies or predicts treatment responses [8,18].

### 5.9. Adaptive and Response-Guided Designs

Adaptive approaches may permit the prespecified modification of recruitment or treatment allocation as evidence accumulates. Such designs require rigorous statistical control and should be distinguished from post hoc subgroup selection.

### 5.10. Challenge Tests and Dynamic Phenotyping

Fasting baseline markers and postprandial dynamic phenotypes provide complementary information. Fasting measurements are useful for characterizing relatively stable metabolic status, including baseline glucose regulation, lipid profile, inflammatory state, and other chronic physiological characteristics. They are therefore appropriate when the objective is to stratify participants according to pre-intervention metabolic state or to develop simple baseline prediction models. In contrast, standardized glucose, mixed-meal, or lipid challenges provide controlled dynamic phenotyping and may reveal differences in metabolic flexibility, glucose and insulin excursions, postprandial lipemia, or other physiological responses that are not apparent under fasting conditions. Studies by Zeevi et al. and Berry et al. demonstrated how repeated postprandial measurements can reveal substantial interindividual metabolic variability that is not apparent from fasting measurements alone [5,6]. Accordingly, fasting and postprandial phenotypes should be selected according to the biological question: fasting markers are generally preferable for stable baseline characterization, whereas challenge-based phenotypes are particularly informative when the aim is to characterize dynamic metabolic responsiveness or resilience.

### 5.11. Matching Clinical Trial Design to the Scientific Question

Population-average efficacy, treatment-response heterogeneity, reproducible individual treatment effects, and prospective response prediction are distinct scientific questions that require increasingly demanding study designs [8,24].

## 6. Biological and Behavioral Determinants of Heterogeneous Responses

### 6.1. Multifactorial Nature of Responses

Interindividual responses reflect interactions among host, behavioral, environmental, and intervention-related factors [6,9,16,17]. No single universal responder phenotype is likely to apply across nutritional outcomes.

### 6.2. Age, Sex, and Baseline Metabolic Status

Age and sex may influence physiology, body composition, hormonal status, and metabolism, whereas baseline nutritional and metabolic status often determines the potential magnitude of improvement [6,9]. However, apparent subgroup differences require formal interaction testing [18,22], and associations between baseline status and changes must be interpreted in light of regression to the mean [2,21].

### 6.3. Genetic Variation

Genetic variations may contribute to heterogeneous dietary responses; however, individual variants often have limited predictive value. In the PREDICT 1 trial, the relative contribution of genetics differed across postprandial outcomes, illustrating that genetic influence is outcome-dependent rather than universally dominant [6].

### 6.4. Gut Microbiome

Gut microbiome composition and function may influence nutrient metabolism, bioavailability, bile acid metabolism, short-chain fatty acid production, and host metabolic responses [5,6,16,17,26]. However, microbiome characteristics are influenced by diet, medication use, geography, and host physiology. Therefore, their predictive value should be demonstrated rather than assumed.

### 6.5. Habitual Diet and Lifestyle

Habitual diet, physical activity, sleep, meal timing, and other behavioral factors may modify treatment responses and contribute to state-dependent variability [5,6,9,16]. These variables may function as confounders of the observed response and as potentially useful predictors.

### 6.6. Medication, Health Status, and Intervention Characteristics

Medication use, comorbidities, dose, intervention duration, formulation, food matrix, and bioavailability may substantially influence observed responses. Therefore, an apparent nonresponse may reflect insufficient exposure or inappropriate intervention conditions rather than intrinsic biological resistance.

### 6.7. Adherence and Exposure

Poor adherence can mimic biological nonresponse. Where feasible, objective exposure biomarkers may complement self-reported adherence, and metabolomics can provide additional information regarding actual dietary exposure [11].

### 6.8. Interactions Among Determinants

Response determinants rarely act independently. Therefore, multivariable approaches integrating clinical, dietary, behavioral, microbiome, and molecular information are often more informative than isolated one-factor subgroup analyses [5,6].

### 6.9. From Candidate Determinants to Validated Predictors

Factors retrospectively associated with responder status should not automatically be considered clinically useful response predictors [18,20]. Candidate modifiers must undergo formal interaction testing or appropriately designed predictive modeling, followed by independent validation.

## 7. Evidence from Functional Food and Dietary Intervention Trials

Representative intervention studies spanning responder classification, biological characterization, baseline prediction, predictive modeling, and external validation are summarized in Table 1.

### 7.1. Omega-3 Fatty Acid Supplementation

Omega-3 supplementation illustrates the potential and limitations of responder analyses. Rundblad et al. defined triglyceride responders using a threshold informed by expected biological variation and subsequently characterized the molecular differences between response groups [27]. This approach offers a stronger rationale for threshold selection over an arbitrary percentage cutoff; however, it primarily remains a post-intervention characterization strategy.

Recent multi-omics analyses have attempted to predict triglyceride responses using baseline molecular information. Morin-Bernier et al. showed that adding additional metabolomic layers did not necessarily improve prediction beyond the existing genomic information [28]. Thus, omega-3 studies illustrate a progression from variability-informed classification toward predictive modeling while also demonstrating that greater molecular complexity does not automatically yield better predictions.

### 7.2. Raspberry and Other Plant-Based Functional Foods

Plant-based intervention studies provide useful examples of post hoc responder phenotyping. Franck et al. used multivariate and transcriptomic approaches to identify distinct response profiles after red raspberry consumption, followed by molecular characterization [29]. Barbe et al. subsequently examined functional microbiome signatures associated with the previously defined responder phenotype [30]. Although these studies provide mechanistic insight, they should be interpreted as biological characterizations because responder groups were derived from the intervention cohort rather than being prospectively predicted and independently validated.

Kim et al. moved closer to prospective predictions by examining baseline urinary metabolites associated with subsequent responses to Korean black raspberry consumption [31]. The decline in predictive performance observed during leave-one-out cross-validation illustrates the importance of accounting for optimism in development datasets.

### 7.3. Probiotic Interventions

Wastyk et al. identified heterogeneous metabolic responses during a probiotic intervention and linked response patterns with habitual diet and microbiome characteristics [32]. Such clustering may generate useful mechanistic hypotheses, but post hoc response subgroups—particularly those following limited or heterogeneous overall effects—require independent confirmation before they can be considered as predictive phenotypes.

### 7.4. Personalized Glycemic Responses

Personalized glycemic-response studies represent a more advanced stage of the evidence continuum. Zeevi et al. demonstrated marked variations in glycemic responses to identical foods and developed a multivariable algorithm incorporating clinical, dietary, behavioral, and microbiome information [5]. Berry et al. extended this approach to multidimensional postprandial glucose, insulin, and triglyceride responses [6], and Mendes-Soares et al. evaluated the transportability of a personalized glycemic-response model in an independent population [7]. These studies are conceptually distinct from conventional responder classification because they estimate continuous individual responses rather than assigning individuals to dichotomous responder categories.

### 7.5. Personalized Dietary Advice and Metabotypes

Kirwan et al. classified individuals according to the extremes of cholesterol change after personalized dietary advice in the Food4Me study and subsequently explored baseline phenotypic predictors [33]. As responder groups were retrospectively derived from the observed response distribution, this study is best interpreted as a post hoc classification followed by exploratory prediction rather than as providing evidence of reproducible individual treatment effects.

O’Sullivan et al. used biochemical and metabolomic clustering to identify a vitamin D-responsive metabotype [34], whereas subsequent metabotype-based intervention studies have attempted to define phenotypes before treatment and test whether those phenotypes stratify the dietary response [35,36]. These studies illustrate the transition from retrospective response characterization toward prospective stratification.

### 7.6. Gastrointestinal Dietary Interventions

Nybacka et al. characterized serum and urinary metabolomic differences between clinically defined responders and non-responders to dietary intervention in irritable bowel syndrome [37]. Wilson et al. extended this concept by examining baseline fecal and urinary metabolites as predictors of subsequent responses to a low-FODMAP diet [38]. Together, these studies clearly illustrate the distinction between explaining an already observed response and predicting that response before intervention.

### 7.7. Emerging Pattern Across Intervention Studies

Across functional food and dietary intervention studies, responder definitions remain heterogeneous, many analyses are retrospective, and independent validation is uncommon [8,9,11]. Nevertheless, the field is gradually progressing along the following evidence continuum: Descriptive heterogeneity → Threshold-based responder classification → Evidence of treatment-response heterogeneity → Biological characterization and candidate predictor identification → Predictive model development → Independent validation → Prospective response-guided intervention. This progression provides the empirical basis for the framework proposed later in this review.

## 8. Metabolomics and Metabotyping for Responder Identification and Prediction

### 8.1. Metabolomics as a Tool for Heterogeneous Responses

Metabolomics provides a dynamic molecular readout integrating dietary exposure, endogenous metabolism, microbial activity, and physiological state [11]. This feature makes metabolomics particularly relevant to precision nutrition, in which prediction may depend on the current physiological state as well as relatively stable individual characteristics.

Importantly, metabolomic biomarkers used in nutritional intervention studies may serve fundamentally different purposes. Exposure or compliance biomarkers indicate whether the intervention was consumed and whether its components or metabolites reached the circulation or other biological compartments, whereas response biomarkers reflect downstream physiological or mechanistic effects of the intervention. For example, circulating phenolic metabolites or alkylresorcinols may provide evidence of dietary exposure or adherence, whereas changes in endogenous lipid, amino acid, or inflammatory metabolic pathways may reflect biological response. This distinction is particularly important in responder analyses because an apparent non-response may result from poor adherence, insufficient absorption, low bioavailability, or interindividual differences in metabolism rather than from a true absence of biological efficacy. Accordingly, exposure and compliance should be evaluated, where feasible, before interpreting an individual as a biological non-responder.

### 8.2. Identification and Biological Explanation

Metabolomics has frequently been used after interventions to characterize individuals exhibiting different clinical responses [31,34,37]. Although such analyses may identify candidate pathways or mechanisms, they should be regarded as explanatory rather than as predictive when response groups are retrospectively defined.

### 8.3. Metabotyping

Metabotyping aims to identify biologically coherent metabolic subgroups that support targeted dietary recommendations [12,13,39]. Unlike post hoc responder classification, metabotypes can, in principle, be defined before intervention and subsequently evaluated as candidate stratification variables. However, metabotype definitions are heterogeneous, and evidence that specific metabotypes reproducibly identify differential treatment responses remains limited [13,36].

Dahal et al. provide an informative example: previously defined metabotypes differed in responses to a glucose challenge, whereas differential responses to a fiber-enriched dietary intervention were not consistently supported after multivariable adjustment [36]. Thus, metabotyping should be regarded as a candidate stratification strategy rather than as providing established evidence of personalized treatment benefits.

### 8.4. Baseline Metabolomic Profiles for Prediction

Baseline metabolomic profiles may provide information regarding subsequent dietary response. Kim et al. identified urinary metabolites associated with later biochemical responses to Korean black raspberry [31], and Wilson et al. reported the potential predictive value of baseline fecal and urinary metabolites for low-FODMAP responses [38]. These studies illustrate the conceptual shift from retrospective explanation toward pre-intervention prediction.

### 8.5. Multi-Omics Analyses Do Not Necessarily Improve Prediction

The inclusion of additional molecular layers does not automatically improve predictive performance. Morin-Bernier et al. showed that adding selected metabolomic variables provided limited improvement over an existing genomic predictor of triglyceride response [28]. Therefore, each additional omics layer should be evaluated according to the incremental predictive value rather than being assumed to improve performance.

### 8.6. Machine Learning and Dynamic Response Prediction

Wang et al. developed a deep-learning framework to predict post-intervention metabolite responses using baseline microbiome, metabolomics, and intervention information across six dietary intervention datasets [40]. Such approaches represent a conceptual shift away from binary responder categories toward continuous, multidimensional response predictions.

### 8.7. Identification → Explanation → Prediction → Validation

Metabolomics-based responder research can be interpreted as a sequence of increasingly demanding objectives: Identification → Explanation → Prediction → Validation. However, these stages should not be conflated. A metabolite that differs between responders and non-responders after intervention is not automatically a pretreatment predictor, and a predictor developed in one dataset is not necessarily generalizable.

### 8.8. Methodological Priorities

Future metabolomics-based responder research should clearly distinguish baseline predictors from posttreatment correlates, control multiple testing, prevent data leakage, incorporate appropriate internal validation, and prioritize independent external validation [11,18,20,28].

## 9. Multi-Omics and Machine-Learning Approaches

### 9.1. Rationale for Multi-Omics Integration

Heterogeneous dietary responses arise from interacting biological systems. Therefore, integrating genomic, metabolomic, transcriptomic, microbiome, clinical, and behavioral data may improve characterization and predictive performance [6,11]. However, biological complexity does not guarantee predictive improvement.

### 9.2. Multi-Omics Responder Phenotyping

Multi-omics studies can provide complementary mechanistic information across molecular layers. For example, Valsesia et al. integrated clinical and multi-omics information to characterize glycemic responders following weight loss [41]. Such analyses are valuable for identifying coordinated biological signatures; however, their utility depends on whether they can be generalized beyond the development dataset.

### 9.3. Machine Learning for Treatment-Response Prediction

Machine-learning methods can model nonlinear and high-dimensional predictor relationships. However, the prediction of an outcome after treatment is conceptually different from that of an individualized treatment effect [18,19]. Individual treatment-effect prediction requires information about the expected outcomes under alternative treatment conditions and appropriate causal assumptions.

### 9.4. Continuous and Multidimensional Prediction

The prediction of continuous or multidimensional responses may preserve more information than binary responder classification does and reduce the dependence on arbitrary thresholds [10,40]. This approach may be particularly advantageous when nutritional effects vary gradually rather than forming distinct biological classes.

### 9.5. Overfitting and Data Leakage

High-dimensional predictive models are particularly vulnerable to overfitting and data leakage. Feature selection, normalization, batch correction, dimensionality reduction, and model tuning should be performed within appropriate resampling procedures instead of using information from the full dataset [20].

### 9.6. Internal and External Validation

Internal cross-validation is necessary but insufficient to establish generalizability. Independent external validation provides stronger evidence that a fixed predictive model is transportable across individuals, populations, and analytical settings [7,18,20]. As a recent example in nutritional therapy, Azulay et al. developed a multi-omics machine-learning model for the response to exclusive enteral nutrition and subsequently evaluated it in an independent prospective cohort [42]. Although this example lies outside conventional functional food intervention, it illustrates the methodological progression from model development toward external validation.

### 9.7. Incremental Value Beyond Simple Clinical Models

Complex predictive models should be compared with simpler models based on routinely available clinical variables. A useful evaluation strategy is to first define a reference clinical model using readily available pre-intervention variables, such as age, sex, body mass index, baseline metabolic markers, habitual diet, or other established predictors, and then assess whether the addition of metabolomic, microbiome, genetic, or other omics data provides meaningful incremental predictive value. This comparison should not rely solely on apparent improvement in discrimination within the development dataset. Incremental value should be evaluated using appropriate measures of predictive performance, including discrimination, calibration, and prediction error, and should be confirmed using internal validation and, ideally, independent external validation. Where prediction is intended to guide intervention decisions, assessment of clinical or practical utility may also be informative. Yeo et al. demonstrated that different biological layers contribute unequally to the prediction of longitudinal weight-loss responses—clinical variables and blood metabolomics provided substantial information, whereas microbial features produced more modest gains and metabolite dynamics did not consistently improve model performance [43]. Therefore, the relevant question is not whether additional omics information is available, but whether it improves prediction beyond a simpler and more readily implementable clinical model.

### 9.8. Interpretability and Biological Plausibility

Biological plausibility can strengthen confidence in a model but does not establish predictive validity. Conversely, highly flexible black-box models may achieve strong apparent performance while providing limited mechanistic interpretation. Therefore, precision nutrition requires a balance among predictive performance, calibration, interpretability, reproducibility, cost, and feasibility.

### 9.9. From Prediction to Clinical Utility

Even an externally validated model is not necessarily clinically or practically useful. The strongest evidence arises when model-guided intervention assignment is prospectively tested against a conventional strategy and improves the relevant outcomes [18,20].

### 9.10. Implications for Precision Nutrition

The use of more predictors does not necessarily produce better predictive performance. Priority should be given to parsimonious, reproducible, and externally validated models whose added complexity provides demonstrable value for intervention selection. As illustrated in Table 1, recent studies have increasingly shifted from molecular characterization toward multivariable prediction and validation, although relatively few have achieved the prospective demonstration of response-guided utility.

**Table 1:** Representative studies investigating heterogeneous responses, responder phenotypes, and response prediction in dietary and functional food interventions.

Study	Intervention/Population	Primary Response Domain	Responder or Prediction Approach	Main Baseline or Molecular Features	Validation Status	Evidence Stage Represented
O’Sullivan et al. (2011) [34]	Vitamin D3 supplementation; adults	Metabolic syndrome-related markers	Post hoc metabolic clustering	Biochemical and metabolomic phenotypes	No independent validation	Biological characterization/responsive metabotype
Zeevi et al. (2015) [5]	Standardized meals and personalized diets; 800 participants	Postprandial glycemic response	Continuous response prediction	Clinical, dietary, behavioral, and gut microbiome variables	Independent cohort plus prospective dietary intervention	Prediction → validation → response-guided intervention
Kirwan et al. (2016) [33]	Personalized dietary advice; Food4Me cohort	Total cholesterol	Extreme quartiles of observed cholesterol changes	Age, baseline cholesterol, glucose, fatty acids, alcohol intake	Internal evaluation only	Post hoc classification → exploratory prediction
Kim et al. (2017) [31]	Korean black raspberry intervention	Oxidative stress and inflammatory biomarkers	Baseline metabolomic prediction of subsequent responses	Urinary metabolites	Leave-one-out cross-validation	Baseline prediction with internal validation
Rundblad et al. (2019) [27]	Omega-3 fatty acid supplementation	Plasma triglycerides	Response threshold based on expected biological variability	PBMC gene expression and lipoprotein characteristics	No independent validation	Variability-informed classification → biological characterization
Mendes-Soares et al. (2019) [7]	Mixed meals; US population	Postprandial glycemic response	Transfer of individualized response model	Clinical and microbiome variables	Independent population validation	External validation/transportability
Berry et al. (2020) [6]	Standardized and free-living meals; PREDICT 1 trial	Postprandial glucose, insulin, and triglycerides	Continuous individualized-response modeling	Genetics, microbiome, meal composition, and clinical and behavioral variables	Large-cohort model validation	Multidimensional response prediction
Valsesia et al. (2020) [41]	Weight-loss intervention	Glycemic response after weight loss	Integrative multi-omics responder modeling	Clinical and multi-omics profiles	Internal/test-set evaluation	Multi-omics characterization → prediction
Nybacka et al. (2021) [37]	Dietary intervention in irritable bowel syndrome	Gastrointestinal symptoms	Clinical responder classification followed by metabolomic profiling	Serum and urinary metabolites	No independent validation	Biological explanation of the responder phenotype
Dahal et al. (2022) [36]	Oral glucose challenge and fiber-enriched intervention	Glucose and metabolic response	Predefined metabotype classification	Baseline metabolic phenotype	Prospective intervention assessment	Prospective evaluation of metabotype stratification
Franck et al. (2022) [29]	Red raspberry consumption	Cardiometabolic outcomes	Multivariate/transcriptomic response clustering	Transcriptomic, lipidomic, and metagenomic characteristics	No independent validation	Post hoc characterization/mechanism generation
Wilson et al. (2023) [38]	Low-FODMAP diet in irritable bowel syndrome	Clinical symptom response	Baseline metabolite prediction	Fecal and urinary metabolites	Internal predictive assessment	Baseline prediction
Wastyk et al. (2023) [32]	Probiotic intervention in adults with metabolic syndrome features	Metabolic and inflammatory outcomes	Longitudinal response clustering	Diet and gut microbiome characteristics	No independent validation	Exploratory response subgroup identification
Morin-Bernier et al. (2024) [28]	Omega-3 fatty acid supplementation	Plasma triglycerides	Multi-omics predictive classification	Genomic, lipidomic, SCFA, and bile acid data	Internal evaluation	Prediction with incremental-value assessment
Wang et al. (2025) [40]	Six dietary intervention datasets	Post-intervention metabolite responses	Continuous multidimensional prediction	Baseline microbiome, metabolome, and intervention information	Multi-dataset evaluation	Machine-learning prediction of dynamic responses
Azulay et al. (2026) [42]	Exclusive enteral nutrition; pediatric Crohn disease	Nutritional therapy response	Multi-omics machine-learning prediction	Serum and fecal metabolomics, lipidomics, and microbiome	Independent prospective validation cohort	Prediction → external validation
Barbe et al. (2026) [30]	Red raspberry intervention	Previously defined cardiometabolic phenotype	Functional microbiome characterization of prior responder groups	Functional gut microbiome signatures	Same/related intervention cohort	Mechanistic characterization

Abbreviations: PBMC, peripheral blood mononuclear cell; SCFA, short-chain fatty acid.

**Table 2:** Common analytical approaches and appropriate interpretation of responder analyses in functional food and dietary intervention trials.

Analytical Approach	Primary Inferential Target	What the Analysis Supports	Key Limitation	Appropriate Role
Single-arm pre–post comparison	Changes over time	Evidence that the measured outcome changed during the observation period	Cannot separate intervention effects from regression to the mean, temporal changes, expectancy, or other uncontrolled factors	Exploratory or early-phase assessment
Separate within-group significance tests in an RCT	Changes within each treatment arm	Description of within-group temporal changes	A significant change in one group and nonsignificant change in another does not demonstrate a treatment difference	Descriptive only; not appropriate for treatment-effect inference
Between-group comparison of changes	Average treatment effects	Difference in the mean change between treatment conditions	Does not establish stable individual treatment effects	Conventional efficacy analysis
Baseline-adjusted ANCOVA	Average treatment effects	Treatment effect adjusted for the baseline outcome	Does not quantify treatment-response heterogeneity	Preferred primary analysis for many parallel-group trials
Treatment × time interaction	Differences in longitudinal trajectory	Whether outcome trajectories differ between treatment groups	Does not identify true individual responders unless individual heterogeneity is explicitly modeled	Longitudinal group-level treatment-effect assessment
Threshold-based responder classification	Crossing a predefined response threshold	Frequency of a prespecified clinically, biologically, or analytically meaningful response	Dichotomization, threshold dependence, and instability near the defined cutoff	Secondary/supportive analysis
Comparison of responder proportions	Group differences in the probability of meeting a threshold	Whether predefined response rates differ between treatments	Does not demonstrate true individual treatment-effect heterogeneity	Supportive categorical treatment comparison
Intervention-versus-control variance comparison	Excess variability attributable to treatment	Preliminary evidence that a treatment response may vary beyond background variability	Sensitive to measurement error, heteroscedasticity, and model assumptions	Screening for potential treatment-response heterogeneity
Repeated baseline/post measurements	Measurement reliability and within-person variation	More precise estimation of biological and analytical variability	Does not provide the counterfactual response to an alternative treatment	Recommended when outcomes show substantial within-person variability
Subgroup-specific treatment estimates	Treatment effects within defined strata	Treatment effect estimates for clinically relevant subgroups	Differences in subgroup-specific statistical significance do not prove effect modification	Descriptive/exploratory unless supported by interaction testing
Treatment × baseline-modifier interaction	Effect modifications	Evidence that treatment effects differ according to a baseline characteristic	Interaction effects often require large samples and may be unstable	Preferred method for testing candidate effect modifiers
Single crossover trial	Within-person treatment contrast	Average treatment differences within participants	One exposure per condition may not establish response reproducibility	Efficient within-person efficacy comparison
Repeated crossover/replicate design	Treatment-by-individual heterogeneity	Separation of within-person variability from reproducible individual treatment differences	Greater burden, duration, and washout requirements	Strong design for evaluating reproducible individual responses
*N*-of-1 trial	Individual causal treatment effects	Reproducible treatment effect within a specific individual	Limited direct population generalizability; requires reversible effects and adequate washout	Individualized treatment-effect estimation
Post hoc responder phenotyping	Biological description of observed response groups	Molecular or clinical characteristics associated with retrospectively defined responders	Association after a response does not constitute prediction	Mechanistic exploration and hypothesis generation
Baseline predictor analysis	Association with future observed responses	Candidate pretreatment predictors of subsequent responses	May overfit and does not establish generalizable prediction without validation	Early-stage predictive research
Multivariable predictive model	Future response prediction	Estimated probability or magnitude of a response before intervention	Internal performance may be optimistic; prediction is not necessarily causal treatment selection	Model development with rigorous internal validation
Multi-omics prediction	Incremental predictive information	Whether integrated biological data improve pretreatment prediction	High dimensionality, cost, batch effects, data leakage, and limited interpretability	Use when incremental value beyond simpler models is demonstrated
Independent external validation	Generalizability	Reproducibility and transportability of a fixed predictive model	Does not demonstrate whether using the prediction improves outcomes	Required before generalizable precision-nutrition claims
Prospective response-guided trial	Clinical or practical utility	Whether predictor-guided intervention selection improves outcomes versus conventional allocation	Requires a validated predictor and feasible treatment-selection strategy	Highest level of evidence for precision-nutrition implementation

Abbreviations: ANCOVA, analysis of covariance; RCT, randomized controlled trial.

## 10. Proposed Framework for Responder Analysis in Functional Food Trials

### 10.1. Rationale for a Structured Framework

Building on the methodological considerations summarized in Table 2 and empirical progression illustrated in Table 1, we propose a seven-level conceptual framework for evidence development in responder research (Figure 1). The central principle is that responder classification should not be the analytical starting point. Investigators should first establish outcome reliability, estimate the treatment effect relative to an appropriate comparator, and determine whether evidence exists for meaningful treatment-response heterogeneity.

### 10.2. Step 1: Define the Response Prospectively

The response outcome, timing, benefit direction, and analytical scale should be defined before examining the treatment results. Generally, the continuous response should be retained as the primary analytical scale. If a categorical threshold is required, its clinical, biological, or analytical rationale should be prespecified whenever possible [10,22].

### 10.3. Step 2: Establish Measurement Reliability

Analytical error and within-person biological variability should be quantified or otherwise considered [2,4,23]. Repeated measurements may be particularly important when the expected nutritional effects are small relative to background variability.

### 10.4. Step 3: Establish the Treatment Effect

Average treatment efficacy should be evaluated using an appropriate comparator and direct between-group inference rather than using separate within-group significance tests [21,22]. Without evidence that the intervention affects the outcome relative to its comparator, the interpretation of responder categories becomes particularly uncertain.

### 10.5. Step 4: Evaluate Treatment-Response Heterogeneity

Investigators should determine whether the observed heterogeneity exceeds that expected from measurement errors and background within-person variations [2,8]. This step distinguishes “Did participants change by different amounts?” from “Is there credible evidence that participants differed in their response to treatment?”

### 10.6. Step 5: Investigate Candidate Modifiers

Biologically plausible baseline characteristics may then be examined as candidate modifiers. Formal treatment-by-modifier interactions should be preferred over comparisons of subgroup-specific statistical significance [18,22]. Exploratory interaction analyses should be clearly identified, particularly in small functional food trials.

### 10.7. Step 6: Separate Identification from Prediction

Responder research should explicitly distinguish Identification—who appeared to respond after the intervention; Explanation—which biological characteristics are associated with the observed response; Prediction—whether information available before treatment predicts a subsequent response; and Validation—whether the prediction can be generalized to new individuals or populations. This distinction is fundamental to the interpretation of omics-based responder research.

### 10.8. Step 7: Develop Predictive Models Conservatively

Predictive models should prioritize parsimony, reproducibility, and appropriate internal validation [20,28]. All data-dependent preprocessing and feature-selection procedures should be incorporated into the validation framework. Complex omics-based models should be compared with simpler clinical models based on routinely available variables, and their incremental predictive value should be demonstrated rather than assumed. Prediction models developed for precision nutrition should also be reported transparently with respect to model development, performance, validation, calibration, discrimination, and intended use. The TRIPOD+AI statement provides updated reporting guidance for prediction model studies using either conventional regression or machine-learning methods [44]. Adherence to such guidance may improve reproducibility, facilitate critical appraisal, and support appropriate interpretation of predictive precision-nutrition models.

### 10.9. Step 8: Validate Independently

External validation should precede claims that a predictor is generalizable or suitable for precision nutrition [7,18,20]. Replication across populations, dietary settings, and analytical platforms is especially important for high-dimensional omics signatures.

### 10.10. Step 9: Test Response-Guided Intervention Prospectively

Ultimately, strong claims of precision-nutrition utility require evidence that using a validated predictor to select an intervention improves outcomes compared with conventional intervention allocation [18,35,45]. Prediction alone is insufficient if it does not improve decision-making. However, prospective response-guided trials are demanding and may not always be immediately feasible. Before making even cautious precision-nutrition inferences, investigators should, at minimum, demonstrate that the relevant treatment-response heterogeneity is reproducible, that candidate predictors are measured before intervention and are biologically or clinically plausible, that predictive performance is internally validated, and that model performance is compared with simpler clinical approaches. Independent external validation is strongly desirable before claims of generalizability. Until prospective response-guided utility has been demonstrated, such findings should be framed as evidence for stratification or prediction rather than as proof that personalized intervention selection improves outcomes.

### 10.11. Proposed Conceptual Levels of Evidence

We propose the following conceptual hierarchy (as illustrated in Figure 1):

- Level 1—Descriptive heterogeneity: variation in observed responses is documented.
- Level 2—Threshold-based responder classification: individuals are classified using a predefined or post hoc response criterion.
- Level 3—Evidence of treatment-response heterogeneity: observed response variability is shown to exceed expected background or control variability.
- Level 4—Biological characterization and candidate predictor identification: biological, clinical, behavioral, or omics-based characteristics associated with heterogeneous treatment responses are identified as candidate modifiers or predictors.
- Level 5—Predictive model development: pre-intervention information is used to predict subsequent individual responses.
- Level 6—Independent validation: predictive performance is reproduced in independent individuals or populations.
- Level 7—Prospective response-guided intervention: a validated predictor is used prospectively to guide intervention selection, and this strategy is shown to improve outcomes compared with conventional allocation.

### 10.12. Recommended Reporting Elements

Responder analyses should report (1) the exact response outcome and timing; (2) whether the response was analyzed continuously, categorically, or both; (3) the rationale and prespecification status of any threshold; (4) measurement reliability and expected within-person variability; (5) the treatment effect relative to an appropriate comparator; (6) whether treatment-response heterogeneity was formally evaluated; (7) whether candidate modifiers were prespecified; (8) treatment-by-modifier interaction analyses; (9) methods used to control multiple testing, overfitting, and data leakage; (10) internal and external validation procedures, model performance including calibration and discrimination, and adherence to relevant prediction-model reporting guidance such as TRIPOD+AI [44]; (11) whether predictors were measured before intervention; and (12) whether prospective response-guided utility was evaluated.

### 10.13. From Classification to Decision Support

The ultimate objective of precision nutrition should not involve the increasingly detailed labeling of individuals as responders and non-responders. Rather, responder research should generate reproducible information that improves intervention selection.

Descriptive heterogeneity → Threshold-based responder classification → Evidence of treatment-response heterogeneity → Biological characterization and candidate predictor identification → Predictive model development → Independent validation → Prospective response-guided intervention.

This progression provides a practical pathway from observed response variability toward scientifically defensible precision nutrition.

## 11. Challenges and Future Directions

### 11.1. Standardization Without Oversimplification

Responder research requires greater consistency in the reporting of response definitions, thresholds, measurement reliability, and validation procedures [3,8,22]. However, standardization should concern methodological principles rather than impose a single universal responder threshold across diverse outcomes.

### 11.2. Reproducibility of Individual Responses

A major unresolved question is whether apparent individual responses are reproducible during repeated exposure. Repeated crossover, replicate, and *N*-of-1 designs can help distinguish stable treatment-by-individual interactions from transient biological variations [1,2,24,25].

### 11.3. External Validation and Transportability

Most responder models are developed in selected cohorts. Future studies should test transportability across different populations, dietary backgrounds, recruitment settings, and analytical platforms [7,18,20]. Multicenter datasets and coordinated validation studies will be particularly important for omics-derived predictors.

### 11.4. Sample Size and Collaborative Data Resources

Studies designed to investigate treatment-effect heterogeneity generally require larger samples than trials designed solely to estimate average effects do [8,18]. When individual studies cannot recruit sufficient participants, pooled individual-participant data or coordinated multicenter analyses may provide more stable estimates.

### 11.5. Dynamic Phenotyping

Traditional baseline and endpoint measurements provide limited information about temporal response patterns. Continuous glucose monitoring, wearables, ambulatory physiological measures, and repeated digital assessments may improve the estimation of within-person variability and reveal dynamic response trajectories [5,6,24].

### 11.6. Integration of Molecular and Behavioral Information

Future predictive models will likely combine relatively stable variables, such as genetics, with dynamic variables, including metabolomics, microbiome function, habitual diet, adherence, sleep, and physical activity [6,11]. Nevertheless, the value of each layer should be assessed according to incremental predictive performance and practical feasibility.

### 11.7. Artificial Intelligence and Model Transparency

Artificial intelligence may improve the modeling of nonlinear and high-dimensional data; however, its value depends on transparent preprocessing, appropriate validation, interpretability, and comparison with simpler models [19,20,40]. Increasing algorithmic complexity should not substitute for sound study design or external validation.

### 11.8. Dynamic Metabolomics and Flux-Based Approaches

Most current metabolomics studies provide static or semi-static snapshots of the metabolic state, whereas the nutritional response is inherently dynamic. Postprandial metabolomics, longitudinal sampling, stable-isotope tracing, and flux-based approaches may provide information on metabolic flexibility and pathway activity beyond static concentrations [11,46]. Therefore, fluxomics may help distinguish individuals with similar baseline metabolite levels but who have different capacities to respond to nutritional challenges [46].

### 11.9. Scalable N-of-1 and Repeated-Intervention Research

Digital technologies may reduce the burden associated with repeated individualized experiments. Aggregated *N*-of-1 and replicate-intervention studies may offer a bridge between rigorous within-person causal estimation and the identification of population-level predictors of individual treatment responses [24].

### 11.10. Regulatory and Health-Claim Considerations

Responder research raises important questions regarding health-claim substantiation. Current health-claim frameworks emphasize evidence of a cause-and-effect relationship between the consumption of a food or constituent and a beneficial physiological effect in the relevant target population [47]. Similar principles apply in other regulatory settings. For example, the U.S. Food and Drug Administration evaluates authorized health claims according to the totality of publicly available scientific evidence and the significant scientific agreement standard [48], while qualified health claims may be permitted when the evidence is supportive but does not meet that standard. Accordingly, exploratory post hoc responder or subgroup findings should not be assumed to provide sufficient evidence for population-level claim substantiation without appropriate confirmatory evidence. Therefore, post hoc responder findings alone would generally provide limited support for a health claim unless the relevant subgroup or predictive strategy is supported by prospectively specified and independently validated evidence. Future regulatory roles of enriched populations, predictive biomarkers, and response-guided intervention designs will require further methodological and policy consideration.

### 11.11. Practical Utility and Cost-Effectiveness

Predictive models must ultimately justify their complexity and cost. For functional foods, where absolute treatment effects may be modest, readily available clinical or dietary variables may offer greater practical value than expensive multi-omics profiling would. Therefore, evaluation of the feasibility, accessibility, cost-effectiveness, and incremental decision value should accompany predictive-performance assessments.

### 11.12. Toward Prospective Precision Nutrition

The field of precision nutrition is gradually shifting from a retrospective description of heterogeneous responses toward prospective intervention selection based on validated predictors. Progress will depend on reliable phenotyping, reproducible evidence of treatment-response heterogeneity, adequately powered prediction studies, independent validation, and prospective demonstrations that personalization improves outcomes. Therefore, the key challenge is not merely to demonstrate that individuals respond differently but also to determine whether those differences can be measured reliably, predicted before intervention, and translated into better intervention decisions. In the absence of prospective response-guided trials, precision-nutrition inferences should therefore remain cautious and should be limited to validated stratification or prediction rather than claims of demonstrated clinical utility.

## 12. Conclusions

Interindividual variability in the response to dietary and functional food interventions is an important consideration in precision nutrition [6,8,9]. However, observed differences in pre–post changes should not automatically be interpreted as evidence of stable responder and non-responder phenotypes [1,2,3,4]. Measurement error, within-person biological variability, regression to the mean, background temporal changes, and arbitrary dichotomization may all generate apparent response heterogeneity [2,3,4,10,21]. Therefore, rigorous evaluation requires a clear distinction between observed outcome variability and true heterogeneity in treatment effects.

Appropriate comparator groups, reliable outcome measurements, interaction analyses, crossover or repeated-intervention designs, and careful assessment of within-person variability become increasingly important as the inferential objective moves beyond the estimation of an average treatment effect [2,4,8,24,25]. Advances in metabolomics, metabotyping, microbiome analysis, multi-omics integration, and machine learning offer new opportunities to characterize and predict heterogeneous responses [11,12,28,31,34,35,40,41,42,43]. Nevertheless, retrospective molecular differences between responders and non-responders should not be regarded as equivalent to prospectively validated response predictors.

Therefore, for functional food research, the relevant progression is from descriptive heterogeneity and post hoc classification toward biological characterization, prospective prediction, independent validation, and response-guided intervention. The proposed framework is intended to clarify the level of evidence required at each stage of this progression and reduce the overinterpretation of apparent responder phenotypes.

Rather than asking simply who appeared to respond, responder research should establish whether the observed differences are reproducible, treatment-specific, predictable before intervention, and actionable in practice. The maturation of precision nutrition will depend on moving beyond retrospective responder labels toward reproducible, externally validated, and prospectively actionable estimates of individual dietary responses.

## Data Availability

No new data were created or analyzed in this study. Data sharing is not applicable to this article.
